# Protective effect and mechanisms of Buyang Huanwu decoction against hypobaric hypoxia-induced brain injury in mice: involvement of inflammatory responses and HIF-1/PI3K-Akt-related pathways

**DOI:** 10.3389/fimmu.2026.1856864

**Published:** 2026-06-29

**Authors:** Zhixing Wang, Bowei Li, Xin Shen, Baoying Shen, Chunqi Yang, Lijun Huang, Hong Cai, Chengcai Lai, Yue Gao

**Affiliations:** 1 School of Traditional Chinese Medicine, Jiangsu Medical College, Yancheng, China; 2 Beijing Institute of Radiation Medicine, Beijing, China; 3 Qinghai University, Xining, China; 4 Chinese PLA Medical School, Chinese PLA General Hospital, Beijing, China; 5 Department of Dermatology, Air Force Medical Center, PLA, Beijing, China; 6 School of Pharmacy, Tianjin University of Traditional Chinese Medicine, Tianjin, China; 7 Faculty of Environment and Life Science, Beijing University of Technology, Beijing, China; 8 School of Pharmacy, Guangdong Pharmaceutical University, Guangzhou, China; 9 State Key Laboratory of Kidney Diseases, Chinese PLA General Hospital, Beijing, China

**Keywords:** brain injury, Buyang Huanwu decoction, HIF-1, hypobaric hypoxia, neuroinflammation, PI3K-Akt signaling

## Abstract

**Background/objectives:**

This study aimed to investigate the preventive and protective effect of Buyang Huanwu Decoction (BHD) against hypobaric hypoxia-induced brain injury in mice and to explore its underlying mechanisms. Particular emphasis was placed on evaluating whether BHD pretreatment could prevent or attenuate hypobaric hypoxia-induced neurological dysfunction and hippocampal injury, and on clarifying its potential mechanisms from the perspectives of inflammatory responses, metabolic regulation, and HIF-1/PI3K-Akt-related pathways.

**Methods:**

A mouse model of hypobaric hypoxia-induced brain injury was established by exposure to a simulated high-altitude hypoxic environment equivalent to an altitude of 6000 m for 72 h. Mice were assigned to the normal control, model, BHD-pretreated, and acetazolamide-positive control groups. BHD and acetazolamide were administered once daily by intragastric gavage, starting 4 days before hypobaric hypoxia exposure and continuing during the 72 h exposure period. Open field testing was performed to assess spontaneous locomotor activity and exploratory behavior. Hippocampal injury was evaluated by hematoxylin and eosin staining, Nissl staining, and HIF-1α immunofluorescence staining. Non-targeted serum metabolomics, network pharmacology, hippocampal transcriptomics, and RT-qPCR validation were integrated to explore the potential mechanisms of BHD pretreatment.

**Results:**

BHD pretreatment prevented hypobaric hypoxia-induced behavioral abnormalities and alleviated hippocampal pathological injury, neuronal loss, and Nissl body reduction. BHD also reduced excessive hippocampal HIF-1α expression. Multi-omics analyses suggested that the protective effect of BHD was associated with the regulation of inflammatory responses, metabolic disturbances, and HIF-1/PI3K-Akt-related signaling. RT-qPCR validation showed that BHD modulated the abnormal expression of HIF-1α, IL-6, VEGFA, NF-κB1, and STX1A in hippocampal tissue.

**Conclusions:**

BHD exerts a preventive neuroprotective effect against hypobaric hypoxia-induced brain injury in mice. Its effects may involve coordinated regulation of hypoxic responses, inflammatory signaling, metabolic remodeling, and HIF-1/PI3K-Akt-related pathways.

## 1 Introduction

Hypobaric hypoxia-induced brain injury is an important experimental pathological basis of high-altitude cerebral injury, and its core pathological processes include reduced oxygen supply, aggravated oxidative stress, amplified neuroinflammation, disruption of the blood-brain barrier, and impairment of neuronal and glial cell function, ultimately leading to structural and functional abnormalities in brain tissue (1, 2). Hypobaric hypoxia not only compromises cerebral energy supply but also induces mitochondrial dysfunction, metabolic substrate reprogramming, and ionic homeostasis imbalance, thereby triggering apoptosis, necrosis, and other forms of programmed cell death. The hippocampus, a critical brain region for learning and memory, is particularly vulnerable to hypoxic stimulation because it is highly dependent on energy metabolism and synaptic plasticity and because its microvascular and neuroimmune microenvironment is more susceptible to inflammatory amplification, endothelial injury, and impaired neurogenesis (3). Previous studies have shown that high-altitude hypoxia can cause hippocampal neuronal loss, disorganized arrangement, increased necrosis or apoptosis, and consequent cognitive dysfunction (4). Meanwhile, hypobaric hypoxia can aggravate oxidative stress, induce blood-brain barrier damage, and amplify neuroinflammatory responses, thereby further exacerbating brain injury (5–8).

Buyang Huanwu Decoction (BHD) is a classical traditional Chinese medicine formula that has been widely discussed in cerebrovascular diseases, including cerebral small vessel disease and stroke-related conditions (9). Previous studies have demonstrated that BHD exerts multi-component, multi-target, and multi-pathway regulatory effects in ischemic brain injury, post-stroke inflammation, angiogenesis, and neuroregeneration. It not only improves neurobehavioral outcomes but also exerts protective effects by modulating oxidative stress, inflammatory responses, pyroptosis, angiogenesis, and synaptic plasticity (10–13). In addition, BHD has been shown to improve hippocampal structure and function, promote the expression of neurotrophic factors, enhance synaptic plasticity, and improve learning and memory ability (14–18). These findings suggest that BHD may have protective potential in the intervention of hypobaric hypoxia-related brain injury.

The HIF-1 and PI3K-Akt pathways play important roles in the onset and progression of high-altitude-related brain injury. HIF-1 is a key transcriptional regulator under hypoxic conditions. After stabilization under low-oxygen conditions, HIF-1α can regulate the expression of genes involved in glycolysis, angiogenesis, and inflammation-related processes; therefore, it exerts both adaptive and pathological effects in brain injury (19, 20). In some studies, upregulation of HIF-1α has been associated with increased neuronal apoptosis and aggravated brain injury (21, 22). The PI3K-Akt pathway is a major cell survival signaling pathway in cerebral ischemic/hypoxic injury and is involved in the regulation of inflammation, oxidative stress, apoptosis, blood-brain barrier homeostasis, and neurovascular unit protection (23–27). Previous evidence has further shown that the PI3K-Akt pathway can interact with the HIF-1-related hypoxic response network and jointly participate in cellular adaptation and repair after brain injury (24). Although a complete experimental evidence chain directly linking BHD to dual regulation of the HIF-1 and PI3K-Akt pathways in hypobaric hypoxia-induced brain injury is still lacking, existing studies indicate that BHD may interfere with hypoxia-related pathological processes by inhibiting inflammation, reducing oxidative stress, promoting angiogenesis, and improving microenvironmental homeostasis (11–18). Therefore, BHD may alleviate hypobaric hypoxia-induced neuroinflammation, blood-brain barrier damage, and hippocampal neuronal injury by regulating HIF-1-related hypoxic responses and PI3K-Akt-related survival signaling networks, thereby exerting a protective effect against high-altitude brain injury (19–27).

Based on this, the present study established a mouse model of hypobaric hypoxia-induced brain injury to evaluate hippocampal damage and behavioral abnormalities under hypobaric hypoxic conditions, as well as the potential protective effect of BHD. Furthermore, by integrating non-targeted serum metabolomics, network pharmacology, and hippocampal transcriptomics, we explored the roles of the HIF-1 and PI3K-Akt signaling pathways during BHD intervention and their relationship with changes in inflammatory responses and differential metabolites.

## 2 Materials and methods

### 2.1 Animals

A total of 48 healthy specific pathogen-free (SPF) male C57BL/6J mice, aged 8 weeks and weighing 18–20 g, were used in this study. The animals were purchased from Beijing Vital River Laboratory Animal Technology Co., Ltd. (license No. SCXK (Jing) 2021-0006). Mice were housed under standard laboratory conditions at 22 ± 2 °C and 50 ± 10% relative humidity, with good ventilation and minimal noise. Animals were maintained under a 12 h light/dark cycle and allowed to acclimatize for 7 days before the experiment. Cages were cleaned daily throughout the study. All procedures were performed in strict accordance with the ethical guidelines for laboratory animal care and were approved by the Experimental Animal Ethics Committee of the Beijing Institute of Radiation Medicine (approval No. IACUC-DWZX-2025-533).

### 2.2 Establishment of the mouse model of hypobaric hypoxia-induced brain injury

To establish the hypobaric hypoxia-induced brain injury model, mice were exposed to a hypobaric hypoxic environment on day 5 of the pretreatment schedule. The hypoxic exposure simulated an altitude of 6000 m and lasted for 72 h. The hypobaric hypoxic conditions were generated using a hypobaric chamber (Shanghai Tawang Intelligent Technology Co., Ltd., Shanghai, China). The chamber pressure was reduced to simulate high-altitude hypobaric hypoxia, and the estimated oxygen partial pressure under this pressure condition was maintained at 7.6–8.1 kPa; oxygen fraction was not independently adjusted. The chamber temperature was maintained at 21–24 °C. This model effectively simulated brain injury under high-altitude hypoxic conditions and provided a reliable experimental platform for the study of high-altitude cerebral injury (HACI) (28, 29).

### 2.3 Preparation of BHD

The BHD formula consisted of Astragali Radix (120 g), Angelicae Sinensis Radix Tail (6 g), Paeoniae Radix Rubra (5 g), Pheretima (3 g), Chuanxiong Rhizoma (3 g), Carthami Flos (3 g), and Persicae Semen (3 g), all of which were purchased from Beijing Tongrentang Runfeng Pharmaceutical Co., Ltd. The composition and ratio of BHD were based on the classical formula and previous pharmacological studies of BHD (9, 31–34). All crude herbal materials were commercially authenticated and supplied with quality-control certificates from the supplier. To improve reproducibility and traceability, the origins and batch numbers of the herbal materials were recorded as follows: Astragali Radix, Inner Mongolia, batch no. 23081101; Angelicae Sinensis Radix Tail, Gansu, batch no. 20240105; Paeoniae Radix Rubra, Inner Mongolia, batch no. 20240321; Pheretima, Guangdong, batch no. 23092701; Chuanxiong Rhizoma, Sichuan, batch no. 23082201; Carthami Flos, Xinjiang, batch no. 20240326; and Persicae Semen, Linyi, Shandong, batch no. 20240529. All herbal materials used in this study were obtained from the same supplier and prepared using the same extraction protocol. The herbal materials were first soaked overnight in 10 volumes of water at room temperature. The mixture was then brought to a boil over high heat for 20 min, followed by decoction over low heat for 1 h. Subsequently, the herbs were extracted twice more with 8 volumes of water each time, using the same boiling and decoction procedure. The three extracts were combined, filtered through three layers of gauze to remove residues, concentrated using a rotary evaporator, and finally lyophilized to obtain the freeze-dried powder, which was stored at −20 °C. The yield of BHD freeze-dried powder was 42%.

### 2.4 Experimental design

**Figure 1:**
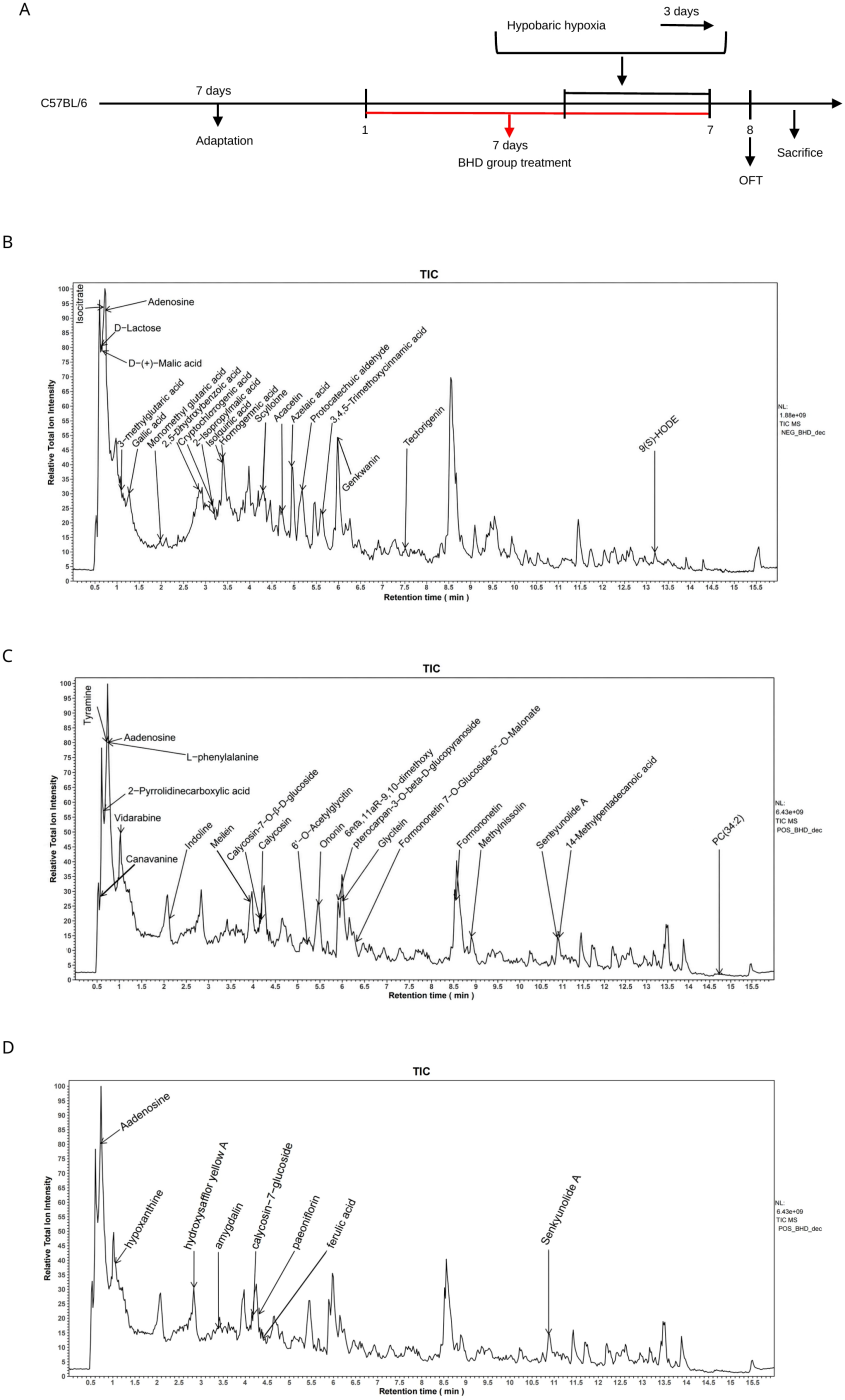
Experimental workflow and LC-MS-based component annotation of Buyang Huanwu Decoction (BHD). **(A)** Schematic diagram of the experimental workflow for mouse pretreatment, hypobaric hypoxia exposure, behavioral testing, and sample collection. **(B)** Total ion chromatogram (TIC) of the BHD sample in negative-ion mode. **(C)** TIC of the BHD sample in positive-ion mode. Major annotated peaks are labeled directly in the chromatograms. **(D)** TIC of the BHD sample with annotated representative constituents used for quality-control assessment, including adenosine, hypoxanthine, hydroxysafflor yellow A, amygdalin, calycosin-7-O-β-D-glucoside, paeoniflorin, ferulic acid, and senkyunolide A. Some representative constituents, such as adenosine, may be annotated in both positive- and negative-ion modes. Compound annotation was based on retention time, accurate mass, MS/MS database matching, and literature-supported chemical information.

Mice were randomly divided into six groups (n = 8 per group): control group (CON), hypobaric hypoxia model group (MOD), acetazolamide pretreatment group (ACZ, 100 mg/kg/day), high-dose BHD group (BHD-H, 7.82 g/kg/day), medium-dose BHD group (BHD-M, 3.91 g/kg/day), and low-dose BHD group (BHD-L, 1.955 g/kg/day). The BHD-H dose was calculated from the conventional adult clinical dose of the crude herbal formula (143 g/day) using body surface area conversion from humans to mice (143 g/70 kg × 9.1) and the freeze-dried powder yield of 42%, resulting in approximately 7.82 g/kg/day of freeze-dried powder. The BHD-M and BHD-L doses were set at one-half and one-quarter of the BHD-H dose, respectively, corresponding to 3.91 and 1.955 g/kg/day. Acetazolamide was used as the positive control because it is an established carbonic anhydrase inhibitor recommended for altitude-related illness (45). The dose of 100 mg/kg/day was selected according to previously reported experimental altitude/hypoxia-related rodent studies using acetazolamide at 100 mg/kg as an intragastric reference intervention (46) and was adapted to the present pretreatment design. Acetazolamide and BHD were administered once daily by intragastric gavage for 7 consecutive days. Specifically, prophylactic administration was initiated 4 days before hypobaric hypoxia exposure and continued during the subsequent 72 h hypobaric hypoxia exposure period. During this period, clinical symptoms were recorded. Following the open field test, mice were deeply anesthetized by intraperitoneal injection of 1% sodium pentobarbital (0.06 mL/10 g body weight, equivalent to 60 mg/kg). After loss of the pedal withdrawal reflex, blood was collected and the mice were euthanized by exsanguination. Death was confirmed by cessation of respiration and heartbeat. Part of the brain tissue was collected for histopathological examination, and the hippocampi were isolated and stored at −80 °C for subsequent analyses. The study design and behavioral testing schedule are shown in **Figure 1A**.

### 2.5 Quality control analysis of BHD

Quality control of the aqueous extract of BHD was performed by LC-MS using a Thermo Scientific UHPLC-Q Exactive ultra-high-performance liquid chromatography-mass spectrometry system. Chromatographic separation was achieved on an ACQUITY UPLC BEH C18 column (100 mm × 2.1 mm, 1.7 μm; Waters, Milford, USA). Mobile phase A consisted of 2% acetonitrile in water containing 0.1% formic acid, and mobile phase B consisted of acetonitrile containing 0.1% formic acid. The injection volume was 3 μL, and the column temperature was maintained at 40 °C. Raw MS data were processed using Progenesis QI v3.0 (Waters Corporation, Milford, USA) for baseline filtering, peak detection, integration, retention time correction, and peak alignment, resulting in a data matrix containing retention time, mass-to-charge ratio, and peak intensity. The representative constituents were putatively annotated using a database-assisted LC-MS workflow based on retention time, accurate mass, isotope/adduct information, MS/MS database matching against a traditional Chinese medicine metabolite database, and literature-supported chemical information. A mass error threshold of <10 ppm was used for MS1 annotation, and MS/MS matching scores were used as supporting evidence. These annotations were used for quality-control assessment rather than absolute standard-compound confirmation.

### 2.6 Open field test

The open field test (OFT), a widely used behavioral paradigm in neuroscience and psychology, was performed to evaluate exploratory behavior, anxiety-like behavior, and locomotor activity (30). The test was conducted in an open field arena measuring 50 × 50 × 35 cm with a white floor. At the start of the test, each mouse was gently placed in the center of the arena and allowed to acclimate for 1 min. Its movement trajectory was then recorded for 5 min using a behavioral tracking system. Parameters including distance traveled in the center zone, percentage of center-zone distance relative to total distance, average speed, and latency to enter the center zone were recorded to assess exploratory activity and anxiety-related behavior.

### 2.7 Immunofluorescence

Before immunofluorescence staining, mouse brain tissues were fixed in 4% paraformaldehyde for at least 24 h. Tissues were then dehydrated through graded ethanol, embedded, and sectioned. Brain sections were immersed in EDTA antigen retrieval buffer (pH 8.0) and subjected to microwave-based antigen retrieval. The tissue area was outlined using a hydrophobic barrier pen to prevent antibody loss. Sections were then treated with an autofluorescence quencher for 5 min, rinsed under running water for 10 min, and blocked with bovine serum albumin (BSA) for 30 min. Rabbit anti-HIF-1α antibody (1:500, Proteintech, Germany GMBH) was applied and incubated overnight at 4 °C, followed by incubation with the secondary antibody at room temperature in the dark for 50 min. Finally, sections were counterstained with DAPI and observed under an upright fluorescence microscope (Nikon Eclipse Ci, Japan). For quantitative analysis, images from the CA1, CA2, CA3, and DG regions were acquired under identical exposure and acquisition settings. HIF-1α fluorescence intensity was quantified using ImageJ by calculating the mean optical density within predefined hippocampal regions of interest. Quantification was performed using three biological replicates per group, and image acquisition and analysis were conducted by investigators blinded to the group allocation.

### 2.8 Histopathological analysis

Brain sections were first dewaxed, followed by hematoxylin staining of nuclei and eosin staining of cytoplasm. After dehydration and mounting, images were acquired using an upright optical microscope (Nikon Eclipse Ci, Japan) to evaluate morphological changes in the hippocampal region. For quantitative analysis, neuronal numbers were counted separately in the CA1, CA2, CA3, and DG regions using fixed microscopic fields under the same magnification. Cells with clear neuronal morphology and visible nuclei were included in the analysis, whereas severely disrupted or indistinct cells were excluded. Three biological replicates per group were analyzed, and all image quantification was performed by investigators blinded to the experimental grouping.

### 2.9 Nissl staining

Dewaxed hippocampal sections were stained with Nissl staining solution (toluidine blue). After staining, the sections were dehydrated, cleared, and cover slipped, and images were captured using an upright optical microscope (Nikon Eclipse Ci, Japan). Nissl-positive neurons/Nissl bodies in the CA1, CA2, CA3, and dentate gyrus (DG) regions of the hippocampus were counted within fixed microscopic fields under the same magnification to evaluate the degree of hippocampal neuronal injury. Three biological replicates per group were analyzed, and quantitative assessment was performed by investigators blinded to the group allocation.

### 2.10 Non-targeted serum metabolomics

#### 2.10.1 Sample collection and preparation

After the behavioral experiments, blood samples were collected from the retro-orbital plexus of mice in each group. Blood samples from the CON, MOD, and BHD-M (3.91 g/kg/day) groups were centrifuged at 12,000 rpm for 15 min at 4 °C. Fifty microliters of serum was transferred to a 1.5 mL EP tube, followed by the addition of 200 μL of cold methanol containing an internal standard. Samples were vortexed for 2 min, incubated at low temperature for 10 min, and centrifuged at 14,000 ×g for 15 min. Then, 200 μL of supernatant was transferred to a new EP tube, freeze-dried or vacuum-concentrated at low temperature, and stored at −20 °C. Before LC-MS analysis, the concentrated metabolite extract was reconstituted in 100 μL of 20% methanol/water, vortexed, centrifuged, and the supernatant was subjected to positive- and negative-ion mode analyses.

#### 2.10.2 LC-MS/MS conditions

LC-MS/MS analysis was performed on an LC-MS/MS system coupled with Waters UPLC BEH C8 and HSS T3 columns. The flow rate was 0.35 mL/min and the injection volume was 5 μL. The mobile phases consisted of 0.1% formic acid in water (A) and 0.1% formic acid in acetonitrile (B). Gradient elution was applied, and data were acquired using full MS scanning combined with data-dependent acquisition (DDA) MS/MS scanning.

#### 2.10.3 Metabolomics data processing and analysis

For non-targeted metabolomics, data were acquired separately in positive- and negative-ion modes, including retention time, MS1, and MS2 information. Blank samples (20% methanol/water) were analyzed first, followed by the actual serum samples. Sample acquisition was performed sequentially to ensure analytical consistency.

### 2.11 Network pharmacology analysis

#### 2.11.1 Collection and screening of BHD targets

Potential targets of Astragali Radix, Angelicae Sinensis Radix, Paeoniae Radix Rubra, Chuanxiong Rhizoma, Carthami Flos, and Persicae Semen in BHD were collected from the Traditional Chinese Medicine Systems Pharmacology Database (TCMSP). The screening criteria were oral bioavailability (OB) ≥ 30% and drug-likeness (DL) ≥ 0.18. Potential targets of Pheretima were further obtained from the BATMAN-TCM database using a score ≥ 20 and P < 0.05 as screening criteria.

#### 2.11.2 Identification of common targets between BHD and high-altitude brain injury

Disease-related targets associated with high-altitude brain injury were collected from the GeneCards and PharmGKB databases. The overlapping targets between BHD and the disease were identified by Venn diagram analysis using R software.

#### 2.11.3 Construction of the PPI network and identification of core targets

A protein-protein interaction (PPI) network was constructed using the STRING database. Core targets were screened using the CytoNCA plugin based on values above the median, and two rounds of screening were performed to identify the final core targets.

#### 2.11.4 GO and KEGG enrichment analyses

GO functional enrichment and KEGG pathway enrichment analyses were performed in R for the common targets shared by BHD and high-altitude brain injury. The top 10 GO terms with P < 0.05 were selected for visualization. Likewise, the top 30 KEGG pathways with P < 0.05 were selected and graphically displayed to elucidate the biological functions and signaling pathways associated with BHD pretreatment.

### 2.12 Transcriptomic analysis

#### 2.12.1 Total RNA extraction from mouse hippocampal tissue

Three hippocampal tissue samples were randomly selected from each of the CON, MOD, and BHD-M groups. Total RNA was extracted using TRIzol reagent. Samples were homogenized using a high-throughput vibration ball mill and lysed at room temperature for 10 min. RNA was then extracted by centrifugation and chloroform separation, purified using magnetic beads and spin columns, and assessed for integrity using an Agilent 2100 Bioanalyzer.

#### 2.12.2 Library construction and RNA sequencing

mRNA was enriched using Oligo(dT) magnetic beads, followed by cDNA synthesis and library construction. Libraries were quantified using a Qubit fluorometer and real-time fluorescence quantitative PCR, and high-throughput RNA sequencing was performed on an Illumina platform.

#### 2.12.3 Basic bioinformatics analysis

Raw sequencing data were processed to remove low-quality reads, and the clean reads were aligned to the reference genome. Differential expression analysis, GO enrichment analysis, and KEGG pathway enrichment analysis were then conducted to identify relevant biological information.

### 2.13 RT-qPCR validation

Total RNA was extracted from frozen mouse hippocampal tissues using TRIzol reagent under RNase-free conditions. Briefly, hippocampal tissues were homogenized with 1 mL of TRIzol reagent using a low-temperature tissue grinder at 30 Hz for 5 min, followed by lysis at room temperature for 10 min. After centrifugation at 12,000 rpm for 5 min at 4 °C, the supernatant was transferred to a new RNase-free tube. Chloroform was then added, and the mixture was vortexed for 15 s, incubated at room temperature for 3 min, and centrifuged at 12,000 rpm for 15 min at 4 °C. The aqueous phase was transferred to a new tube, mixed with an equal volume of isopropanol, and incubated at room temperature for 10 min. RNA was precipitated by centrifugation at 12,000 rpm for 10 min at 4 °C, washed with 75% ethanol, air-dried, and dissolved in RNase-free water. RNA concentration and purity were assessed using a microvolume spectrophotometer, and samples with an OD260/280 ratio of 1.8-2.0 were used for subsequent analysis. cDNA was synthesized using TransScript One-Step gDNA Removal and cDNA Synthesis SuperMix (TransGen Biotech, Beijing, China) according to the manufacturer’s instructions. The reverse transcription reaction was performed at 25 °C for 10 min, 42 °C for 15 min, and 85 °C for 5 s, and the resulting cDNA was stored at -20 °C until use. RT-qPCR was performed using TransStart Green qPCR SuperMix (TransGen Biotech, Beijing, China) in a 10 μL reaction system containing 0.2 μL of forward primer, 0.2 μL of reverse primer, 5 μL of 2× TransStart Green qPCR SuperMix, 0.2 μL of Passive Reference Dye, 1 μL of cDNA, and 3.4 μL of RNase-free water. The amplification conditions were as follows: initial denaturation at 94 °C for 30 s, followed by 40 cycles of denaturation at 94 °C for 5 s, annealing at 55 °C for 15 s, and extension at 72 °C for 30 s. GAPDH was used as the internal reference gene, and relative gene expression was calculated using the 2^(-ΔΔCt) method. For RT-qPCR validation, four independent hippocampal samples obtained from four individual mice were analyzed in each experimental group; therefore, n = 4 biological replicates per group. Primer sequences are provided in **Supplementary Table 2**.

### 2.14 Statistical analysis

**Figure 2:**
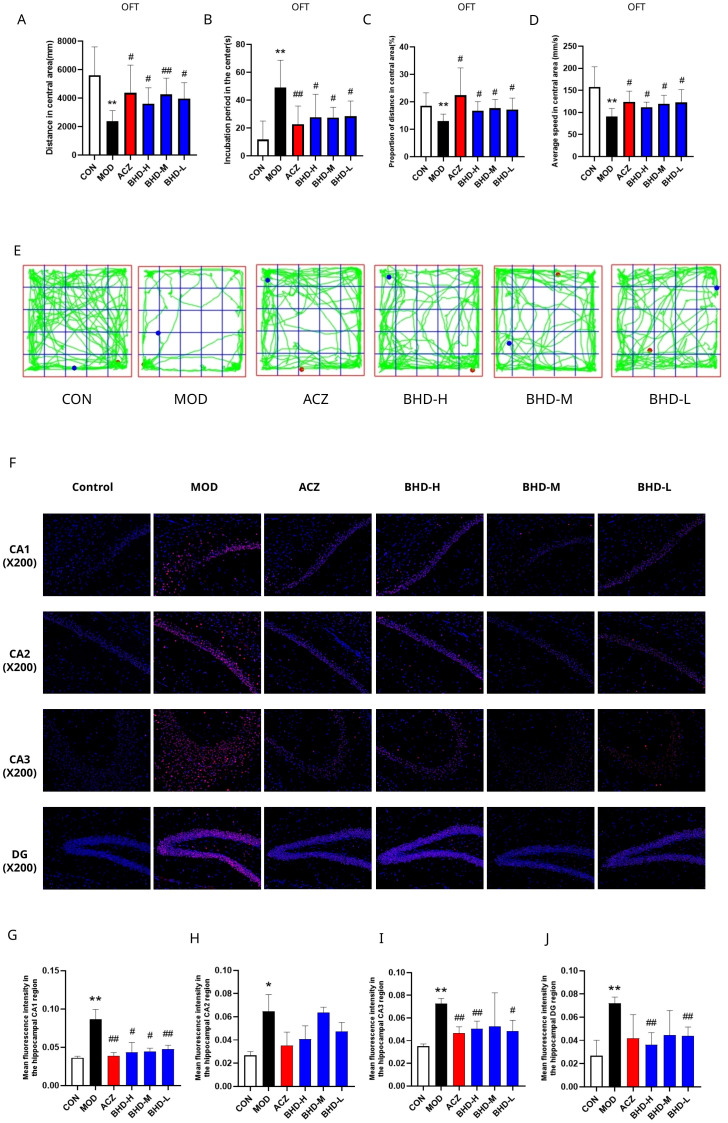
Effects of BHD pretreatment on behavioral deficits and hippocampal HIF-1α expression in mice exposed to hypobaric hypoxia. **(A)** Distance traveled in the center zone in the OFT. **(B)** Latency to enter the center zone in the OFT. **(C)** Percentage of center-zone distance in the OFT. **(D)** Average speed in the OFT. **(E)** Representative movement trajectories in the OFT. Red dots indicate starting points, blue dots indicate endpoints, and green lines represent locomotor tracks (n = 8). **(F)** Representative immunofluorescence images of HIF-1α in the CA1, CA2, CA3, and DG regions of the hippocampus (×200, scale bar = 50 μm). Blue indicates DAPI-stained nuclei, and red indicates HIF-1α expression (n = 3). **(G–J)** Mean optical density of HIF-1α in the CA1, CA2, CA3, and DG regions. Groups included CON, MOD, ACZ, BHD-H, BHD-M, and BHD-L. Data are presented as mean ± SD. *P < 0.05 and **P < 0.01 vs. CON; #P < 0.05 and ##P < 0.01 vs. MOD.

Statistical analyses were performed using GraphPad Prism 8.0.2 software. For normally distributed data, one-way analysis of variance (ANOVA) followed by Tukey’s multiple-comparisons test was used for comparisons among groups. Because of the relatively small sample size, RT-qPCR data were analyzed using the Kruskal–Wallis test followed by Dunn’s multiple-comparisons test. A value of P < 0.05 was considered statistically significant. Unless otherwise specified, data are presented as mean ± SD. For RT-qPCR validation in **Figure 2D**, data are presented as box plots with individual data points; the center line indicates the median, the boxes indicate the interquartile range, and the whiskers indicate the minimum and maximum values.

## 3 Results

### 3.1 Identification of the main chemical constituents of BHD

Calycosin-7-O-β-D-glucoside, ferulic acid, paeoniflorin, senkyunolide A, hydroxysafflor yellow A, amygdalin, hypoxanthine, and adenosine were selected as representative reported constituents for component annotation and quality-control assessment of BHD (31–34), and their retention times are listed in **Table 1**. Total ion chromatograms (TICs) showed that multiple chemical constituents could be detected in the aqueous extract of BHD under both positive- and negative-ion modes, indicating a complex chemical basis for this formula. Major annotated peaks were labeled directly in the chromatograms in both ionization modes (**Figures 1B, C**). In addition, a representative annotated TIC panel was used to clearly show the selected constituents for quality-control assessment, including adenosine, hypoxanthine, hydroxysafflor yellow A, amygdalin, calycosin-7-O-β-D-glucoside, paeoniflorin, ferulic acid, and senkyunolide A (**Figure 1D**; **Table 1**). Compound annotation was based on retention time, accurate mass, MS/MS database matching, and literature-supported chemical information. These results indicate that the BHD aqueous extract prepared in this study contained representative chemical constituents derived from the major herbal components of the formula, supporting the suitability of the preparation for subsequent pharmacodynamic and mechanistic studies.

**Table 1:** Representative annotated compounds used for quality-control assessment of Buyang Huanwu Decoction and their retention times.

Annotated compound	Retention time (min)	Herbal source
Calycosin-7-O-β-D-glucoside	4.19	Astragali Radix
Ferulic acid	4.30	Angelicae Sinensis Radix/Chuanxiong Rhizoma
Paeoniflorin	4.33	Paeoniae Radix Rubra
Senkyunolide A	10.88	Chuanxiong Rhizoma
Hydroxysafflor yellow A	2.85	Carthami Flos
Amygdalin	3.39	Persicae Semen
Hypoxanthine	1.08	Pheretima
Adenosine	0.72	Pheretima

Calycosin-7-O-β-D-glucoside, ferulic acid, paeoniflorin, senkyunolide A, hydroxysafflor yellow A, amygdalin, hypoxanthine, and adenosine were selected as representative reported constituents for LC-MS-based component annotation and quality-control assessment of BHD. Compound annotation was based on retention time, accurate mass, MS/MS database matching, and literature-supported chemical information.

### 3.2 Effects of BHD on behavioral deficits and hippocampal HIF-1α expression in mice with hypobaric hypoxia-induced brain injury

The OFT was used to evaluate behavioral changes in mice with hypobaric hypoxia-induced brain injury. Compared with the control group, mice in the model group showed significantly reduced distance traveled in the center zone, percentage of center-zone distance, and average speed, together with significantly prolonged latency to enter the center zone, indicating that hypobaric hypoxia exposure markedly suppressed spontaneous locomotor activity and exploratory behavior (**Figures 2A–D**). Compared with the model group, BHD pretreatment markedly improved center-zone distance, center-zone distance percentage, and average speed, while significantly shortening center-zone latency, suggesting that BHD effectively reversed the behavioral deficits induced by hypobaric hypoxia (**Figures 2A–E**).

To further investigate the effect of hypobaric hypoxia on hippocampal HIF-1α expression, immunofluorescence staining was performed. The results showed that HIF-1α expression was significantly increased in the CA1, CA2, CA3, and DG regions of the hippocampus in model mice. After BHD pretreatment, HIF-1α expression was markedly reduced in the CA1, CA3, and DG regions (**Figures 2F–J**). As a key transcription factor involved in hypoxic responses, HIF-1α helps regulate multiple biological processes under hypoxic conditions; although it may transiently facilitate oxygen adaptation, persistent overactivation can have detrimental effects (35, 36). The present findings suggest that BHD alleviated hypobaric hypoxia-induced hippocampal injury, at least in part, by suppressing the excessive expression of HIF-1α.

Taken together, the hypobaric hypoxia-induced brain injury model was successfully established, and BHD pretreatment attenuated behavioral deficits and inhibited excessive HIF-1α expression in the hippocampus, indicating a protective effect against hypobaric hypoxia-induced neural injury.

### 3.3 Effects of BHD on hippocampal morphological changes induced by hypobaric hypoxia

**Figure 3:**
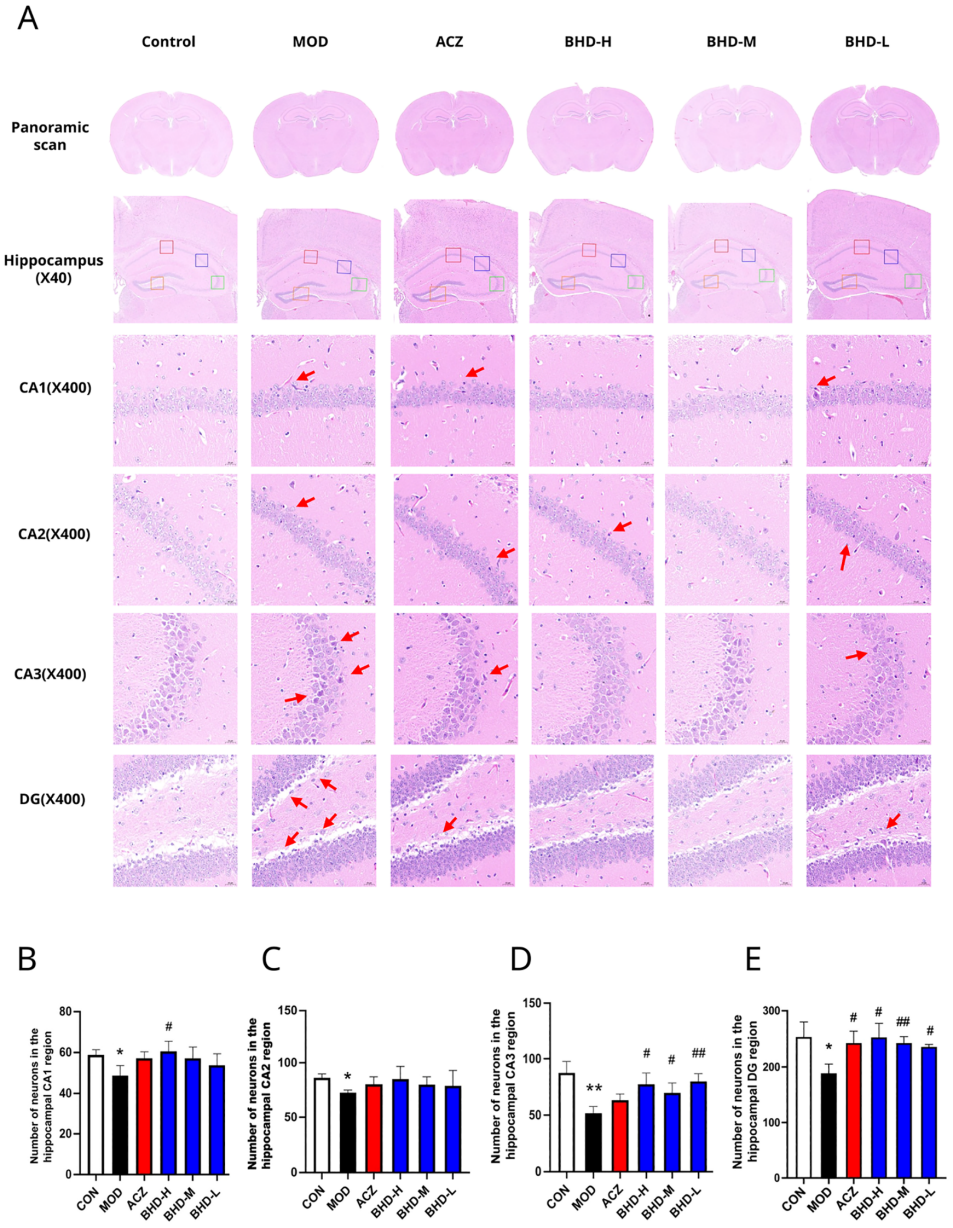
Effects of BHD pretreatment on hippocampal morphology in mice exposed to hypobaric hypoxia. **(A)** Representative H&E staining images of the hippocampus. Images of the CA1, CA2, CA3, and DG regions are shown at ×400 magnification (scale bar = 20 μm). Red, blue, green, and orange boxes indicate the CA1, CA2, CA3, and DG regions, respectively. **(B–E)** Quantitative analysis of neuronal number in the CA1, CA2, CA3, and DG regions; the vertical axes are labeled as **“**Number of neurons**”**. Data are presented as mean ± SD (n = 3). *P < 0.05 and **P < 0.01 vs. CON; #P < 0.05 and ##P < 0.01 vs. MOD.

H&E staining was used to evaluate morphological changes in the hippocampus after hypobaric hypoxia exposure (**Figure 3A**). Compared with the control group, the number of neurons in the CA1, CA2, CA3, and DG regions was markedly reduced in the model group (**Figures 3B–E**). Mild structural abnormalities were observed in the hippocampal tissue, with partial neuronal degeneration, deeper staining, and an indistinct cytoplasm. In contrast, the hippocampal structure in the BHD-pretreated groups was markedly improved, as indicated by lighter staining, better-preserved architecture, and more orderly cellular arrangement.

In the CA1 region, the number of neurons in the model group was significantly lower than that in the control group, whereas the high-dose BHD group recovered to nearly normal levels (**Figure 3B**). In the CA2 region, the improvement in neuronal number after BHD pretreatment was relatively limited and did not reach significance (**Figure 3C**). In the CA3 region, neuronal loss was pronounced in the model group, whereas high-dose BHD significantly suppressed neuronal loss and showed superior efficacy compared with low-dose BHD and ACZ (**Figure 3D**). In the DG region, neuronal numbers were significantly reduced in the model group; recovery in the ACZ group was limited, whereas all BHD dose groups significantly improved neuronal numbers, with the high-dose group showing the best effect (**Figure 3E**). Overall, BHD, particularly at the high dose, significantly alleviated neuronal injury in the CA1, CA3, and DG regions induced by hypobaric hypoxia, indicating a pronounced neuroprotective effect in repairing hypoxic brain injury.

### 3.4 Effects of BHD on Nissl bodies in the hippocampus of mice exposed to hypobaric hypoxia

**Figure 4:**
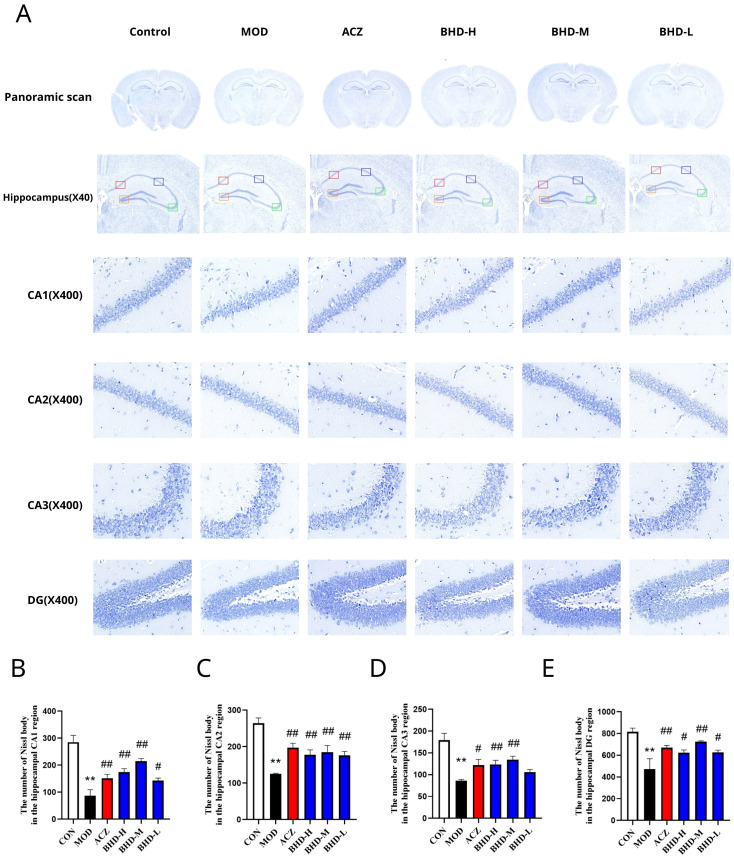
Effects of BHD pretreatment on hippocampal Nissl bodies in mice exposed to hypobaric hypoxia. **(A)** Representative Nissl staining images of the hippocampus. Images of the CA1, CA2, CA3, and DG regions are shown at ×400 magnification (scale bar = 20 μm). Red, blue, green, and orange boxes indicate the CA1, CA2, CA3, and DG regions, respectively. **(B–E)** Quantitative analysis of Nissl-positive neurons/Nissl bodies in the CA1, CA2, CA3, and DG regions. Data are presented as mean +/- SD (n = 3). **P < 0.01 vs. CON; #P < 0.05 and ##P < 0.01 vs. MOD.

Nissl staining showed that, compared with the control group, the number of Nissl bodies in the CA1, CA2, CA3, and DG regions of the hippocampus was markedly reduced in the model group, indicating that hypobaric hypoxia exposure induced hippocampal neuronal injury and loss of Nissl substance (**Figures 4A–E**). Compared with the model group, BHD pretreatment increased the number of Nissl bodies in all hippocampal regions to varying degrees, suggesting a protective effect against hypobaric hypoxia-induced neuronal damage. Among the pretreatment groups, the BHD-M group showed relatively more pronounced improvement in the CA1, CA3, and DG regions, whereas all intervention groups exhibited a broadly consistent recovery trend in the CA2 region. Overall, BHD partially prevented the hypobaric hypoxia-induced reduction in hippocampal Nissl bodies and alleviated neuronal structural damage.

### 3.5 Effects of BHD on serum metabolites in mice with hypobaric hypoxia-induced brain injury

**Figure 5:**
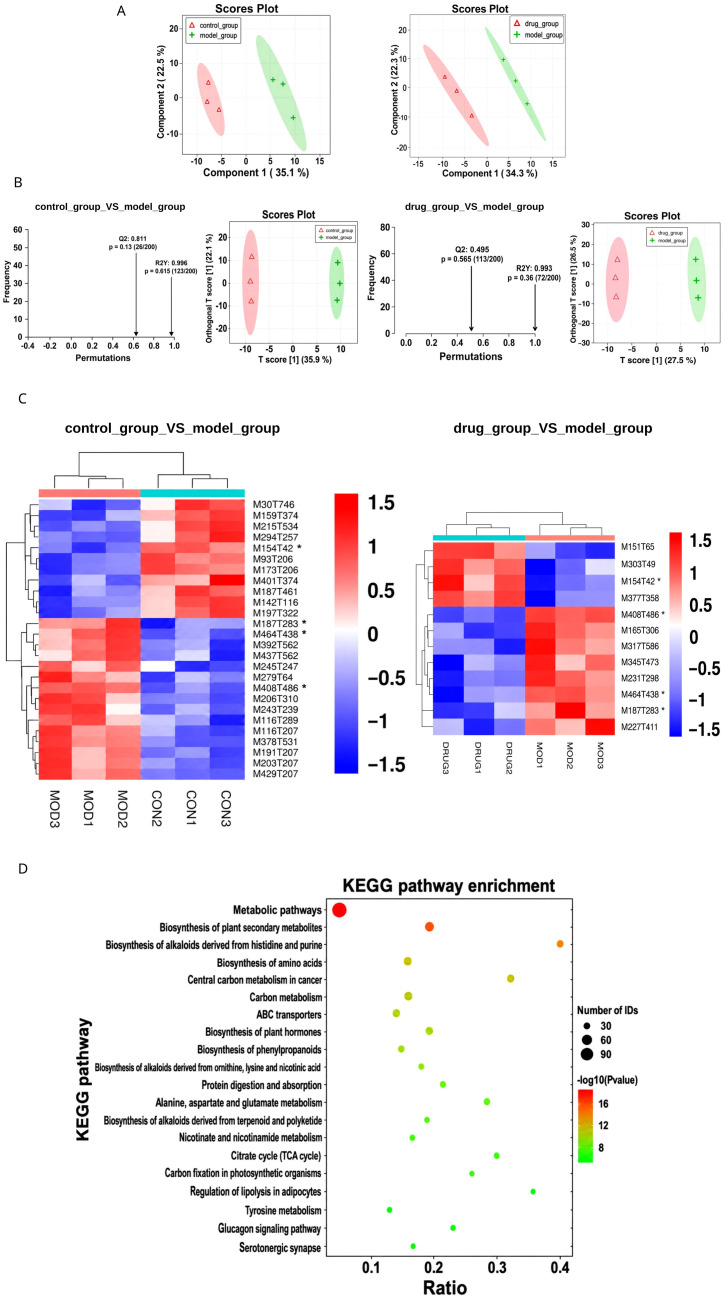
Non-targeted serum metabolomics analysis of BHD-M intervention in mice with hypobaric hypoxia-induced brain injury. **(A)** PLS-DA score plots in negative-ion mode comparing the control and model groups and the BHD-M and model groups. **(B)** OPLS-DA validation plots and permutation testing in negative-ion mode. **(C)** Heatmap-based hierarchical clustering of differential metabolites in negative-ion mode. Metabolites are displayed using Feature IDs to avoid overcrowding. **(D)** KEGG pathway enrichment analysis of differential metabolites. The four main reversed metabolites are annotated as follows: M154T42, L-histidine; M187T283, p-cresol sulfate; M408T486, hyocholic acid; and M464T438, glycocholic acid. Additional Feature ID annotations shown in panel **(C)** include the following: M381T746, cortexolone; M159T374, 3-hydroxyoctanoic acid; M215T534, 3-hydroxydodecanoic acid; M227T411, metyrapol; M93T206, phenol; M173T206, phenyl sulfate; M401T374, trandolaprilat; M187T461, Nϵ,Nϵ,Nϵ-trimethyllysine; M142T116, 2-aminoadipic acid; M197T322, 3,4-dihydroxy-L-phenylalanine; M392T562, chenodeoxycholic acid; M437T562, deoxycholic acid; M245T247, γ,γ-dimethylallyl pyrophosphate ammonium salt; M279T64, uridine; M206T310, 4-(methylnitrosamino)-1-(3-pyridyl)-1-butanone; M243T239, D-biotin; M116T289, indole; M116T207, ketovaline; M378T531, sphingosine-1-phosphate; M191T207, citric acid; M203T207, L-tryptophan; M429T207, L-tryptophan; M151T65, xanthine; M303T49, inosine; M377T358, trehalose; M165T306, phenyllactic acid; M317T586, prostaglandin A1; M345T473, corticosterone; and M231T298, phenobarbital. Supporting metabolomic correlation and classification analyses are provided in **Supplementary Figure 1**.

Non-targeted metabolomics analysis was performed using serum samples collected from the control, model, and BHD-M groups. The PLS-DA score plots showed clear separation between the control and model groups, as well as between the model and BHD-M groups (**Figure 5A**), indicating distinct metabolic profiles and good model discrimination. OPLS-DA analysis and permutation testing (**Figure 5B**) further demonstrated that, in negative-ion mode, both comparisons yielded satisfactory R2Y and Q2 values, indicating good predictive performance of the model.

Differential metabolite analysis in negative-ion mode revealed one upregulated and two downregulated metabolites in the model group compared with the control group. In the BHD-M group, eight metabolites were upregulated and twelve were downregulated. To improve figure readability, metabolite labels in **Figure 5C** are displayed as Feature IDs, and the corresponding metabolite annotations are provided in the **Figure 5** legend and **Supplementary Table 1**. Notably, BHD significantly reversed hypobaric hypoxia-induced changes in several representative metabolites, including M154T42 (L-histidine), M187T283 (p-cresol sulfate), M408T486 (hyocholic acid), and M464T438 (glycocholic acid) (**Figure 5C**). Representative LC-MS/MS annotation information for selected differential metabolites, including L-histidine, p-cresol sulfate, hyocholic acid, and glycocholic acid, is provided in **Supplementary Table 1**. Hierarchical clustering of differential metabolites (**Figure 5C**) showed that the metabolic profile of the BHD-M group was clearly distinct from that of the model group and was closer to that of the control group, further supporting the reliability of differential metabolite screening.

Correlation analysis and metabolite classification results are provided in **Supplementary Figure 1**. The correlation matrices of differential metabolites are shown in **Supplementary Figure 1A**. HMDB and LMSD classification indicated that the differential metabolites were mainly associated with lipids, organic acids, phenylpropanoids, steroids, bile acids, glycerophospholipids, and fatty acyls (**Supplementary Figure 1B**). KEGG classification further showed that these metabolites were mainly involved in metabolic and organismal-system-related categories (**Supplementary Figure 1C**). KEGG pathway enrichment analysis (**Figure 5D**) suggested that these metabolites were closely related to pathways involved in metabolic regulation and nervous-system-related processes. In particular, significant enrichment in central carbon metabolism in cancer and amino acid biosynthesis suggested that BHD exerts substantial metabolic regulatory effects, which may underlie its protective potential in hypoxia-induced brain injury.

### 3.6 Network pharmacology revealed the potential targets and pathways of BHD against hypobaric hypoxia-induced brain injury

**Figure 6:**
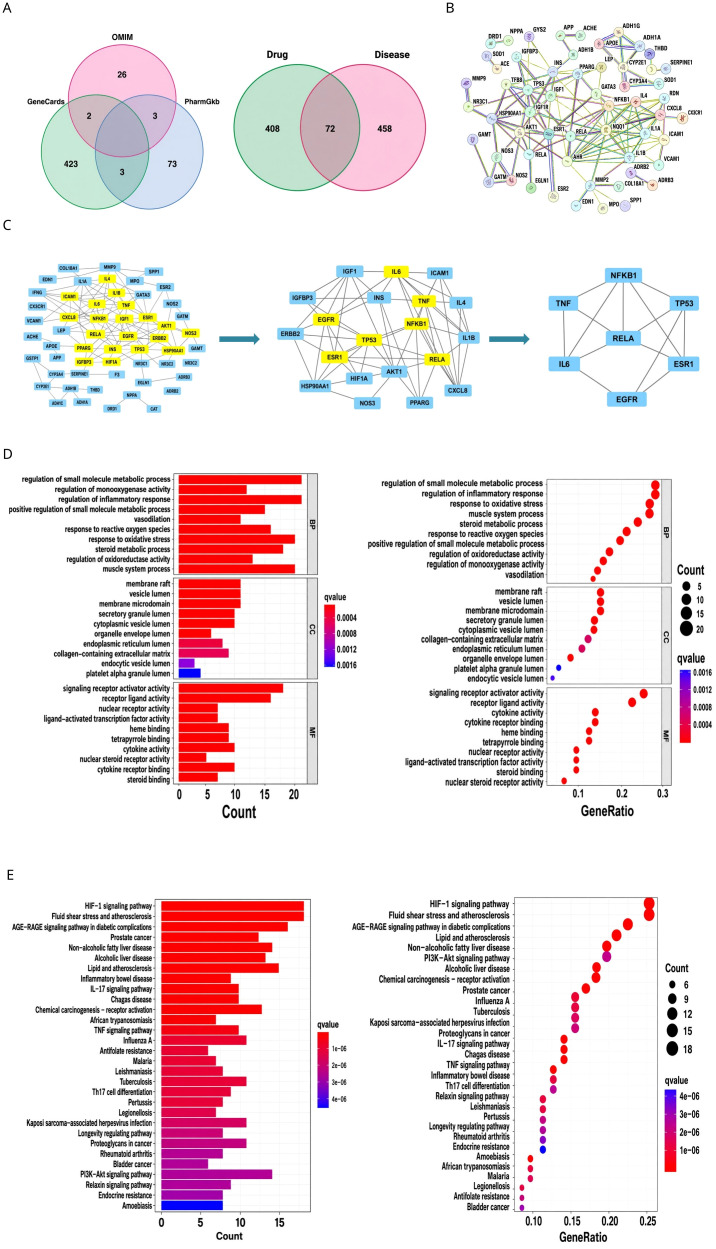
Network pharmacology analysis predicting the potential components, targets, and pathways of BHD against hypobaric hypoxia-induced brain injury. **(A)** Venn diagram showing the overlap between BHD-related targets and high-altitude brain injury-related genes. **(B)** PPI network of common targets. **(C)** Identification of core nodes in the PPI network. **(D)** GO enrichment results presented as bar plots and bubble plots. **(E)** KEGG enrichment results presented as bar plots and bubble plots. Bar length and bubble size represent the number of enriched targets, while the color gradient from red to blue indicates P-value ranking, with deeper red indicating greater pathway significance.

To identify potential protective targets of BHD against hypobaric hypoxia-induced brain injury, targets associated with high-altitude brain injury were collected from GeneCards, PharmGKB, and other databases, yielding a total of 514 candidate targets (**Figure 6A**). Venn analysis identified 72 overlapping targets shared between BHD and high-altitude brain injury (**Figure 6A**). The protein–protein interaction (PPI) network constructed from these common targets suggested potential protective targets of BHD, and further analysis using the CytoNCA plugin identified TNF, EGFR, IL6, TP53, and NF-κB1 as core targets (**Figures 6B, C**). These core targets may play crucial roles in the protective effects of BHD on hypobaric hypoxia-induced brain injury.

GO enrichment analysis revealed that BHD may primarily affect extracellular matrix organization, membrane-related components, and immune responses. More specifically, the protective effects of BHD were mainly associated with extracellular structure organization, the external side of the plasma membrane, and signal transduction-related functions (**Figure 6D**). KEGG pathway enrichment analysis showed that BHD pretreatment involved multiple signaling pathways, particularly the HIF-1 signaling pathway, AGE-RAGE signaling pathway, and pathways associated with cell proliferation and immune responses (**Figure 6E**). These findings suggest that BHD may prevent or attenuate hypobaric hypoxia-induced brain injury by regulating key targets such as TNF, EGFR, IL6, TP53, and NF-κB1, thereby exerting significant neuroprotective effects.

### 3.7 Transcriptomic characteristics of hippocampal tissue after BHD intervention and RT-qPCR validation

**Figure 7:**
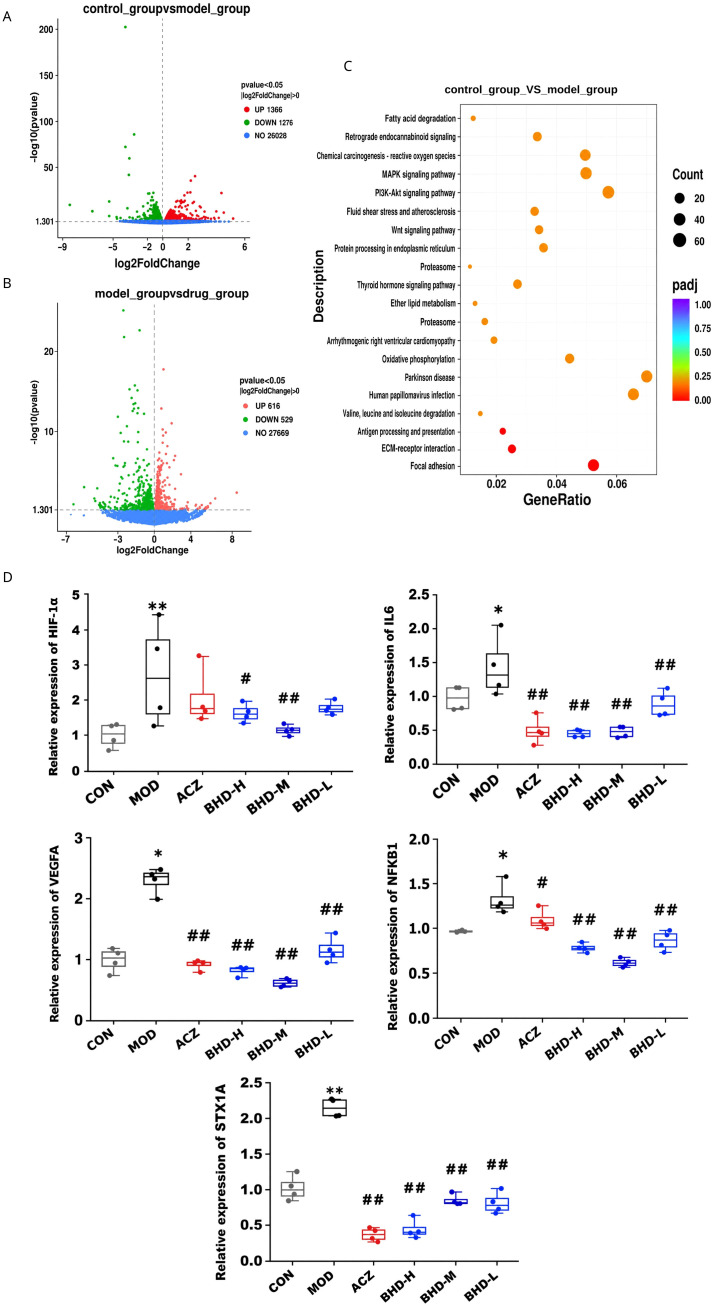
Effects of BHD-M intervention on hippocampal transcriptomic characteristics and key gene expression in mice exposed to hypobaric hypoxia. **(A)** Volcano plot of differentially expressed genes between the CON and MOD groups. **(B)** Volcano plot of differentially expressed genes between the MOD and BHD-M groups. **(C)** KEGG enrichment analysis of differentially expressed genes in the CON vs. MOD comparison. **(D)** RT-qPCR validation of HIF-1α, IL-6, VEGFA, NF-κB1, and STX1A expression in hippocampal tissues. GO enrichment analysis and additional KEGG enrichment results are provided in **Supplementary Figure 2**. Groups included CON, MOD, ACZ, BHD-H, BHD-M, and BHD-L. RT-qPCR data are presented as box plots with individual data points. The center line indicates the median, the boxes indicate the interquartile range, and the whiskers indicate the minimum and maximum values. n = 4 biological replicates per group, with each biological replicate representing an independent hippocampal sample from one individual mouse. RT-qPCR data were analyzed using the Kruskal–Wallis test followed by Dunn**’**s multiple-comparisons test. *P < 0.05 and **P < 0.01 vs. CON; #P < 0.05 and ##P < 0.01 vs. MOD.

To further elucidate the molecular mechanisms underlying the protective effect of BHD against hypobaric hypoxia-induced brain injury, RNA-seq was performed using hippocampal tissues from the CON, MOD, and BHD-M groups. Differential expression analysis showed that, compared with the control group, the model group had 1366 upregulated genes and 1276 downregulated genes; compared with the model group, the BHD-M group had 616 upregulated genes and 529 downregulated genes (**Figures 7A, B**). These findings indicate that hypobaric hypoxia exposure markedly remodeled the hippocampal transcriptome, whereas BHD-M intervention partially reversed this abnormal transcriptional profile.

GO enrichment analysis showed that the differentially expressed genes were mainly associated with inflammatory responses, oxidative stress, and small-molecule metabolic regulation (**Supplementary Figures 2A, B**). In terms of cellular components, these genes were enriched in membrane rafts, vesicle lumen, and collagen-containing extracellular matrix, while molecular functions mainly involved receptor–ligand activity and cytokine receptor binding. KEGG analysis revealed that, in the comparison between the control and model groups, differential genes were mainly enriched in Focal adhesion, ECM-receptor interaction, Antigen processing and presentation, PI3K-Akt signaling pathway, MAPK signaling pathway, and Fluid shear stress and atherosclerosis (**Figure 7C**). In the comparison between the model and BHD-M groups, enriched pathways included Glutamatergic synapse, Antigen processing and presentation, Epstein–Barr virus infection, Cholinergic synapse, Circadian entrainment, and Insulin secretion (**Supplementary Figure 2C**). Notably, the PI3K-Akt signaling pathway was enriched in both the prior network pharmacology analysis and the transcriptomic KEGG analysis, suggesting that this pathway may be an important molecular basis for BHD intervention in hypobaric hypoxia-induced brain injury. Combined with the abnormal increase in HIF-1α under hypobaric hypoxia and its close interaction with PI3K-Akt signaling, these findings further suggest that hypobaric hypoxia-induced hippocampal injury may be associated with dysregulation of the HIF-1-related hypoxic response network, which could be modulated by BHD-M.

Based on the results of network pharmacology, metabolomics, and transcriptomics, VEGFA and STX1A were selected as transcriptome-derived candidate targets, while HIF-1α, IL-6, and NF-κB1 were selected as genes related to hypoxic responses and core targets identified by network pharmacology for RT-qPCR validation (**Figure 7D**). Compared with the control group, mRNA expression levels of HIF-1α, IL-6, VEGFA, NF-κB1, and STX1A in the hippocampus were upregulated in the model group. Compared with the model group, BHD pretreatment downregulated the expression of these genes to varying degrees. Specifically, HIF-1α expression was decreased after BHD pretreatment, with a more pronounced reduction observed in the BHD-M group; IL-6 and VEGFA were decreased in all BHD dose groups; NF-κB1 showed a downward trend in the BHD-H, BHD-M, and BHD-L groups, with the greatest reduction in the BHD-M group; and STX1A was downregulated in the ACZ group and in all BHD dose groups. Overall, the RT-qPCR results were broadly consistent with the trends revealed by network pharmacology and transcriptomics, further supporting the possibility that BHD exerts neuroprotection by modulating hypoxic responses, inflammatory reactions, and synaptic dysfunction, potentially through the HIF-1/PI3K-Akt-related signaling network.

## 4 Discussion

Hypobaric hypoxia-induced brain injury is an important pathological basis of high-altitude cerebral injury and is essentially characterized by cerebral hypoxia, impaired energy metabolism, blood-brain barrier disruption, activation of neuroinflammation, and secondary neuronal damage (2). Previous studies have shown that exposure to high-altitude hypoxia or hypobaric hypoxia significantly aggravates brain histopathological injury and is accompanied by behavioral abnormalities, enhanced oxidative stress, blood-brain barrier disruption, and cerebral metabolic imbalance (3–8, 37, 38). In the present study, hypobaric hypoxia exposure led to marked reductions in spontaneous locomotor activity and exploratory behavior, accompanied by decreased numbers of hippocampal neurons and Nissl bodies, as well as increased HIF-1α expression, indicating that hypobaric hypoxia induces not only neurological dysfunction but also substantial hippocampal injury. Following BHD pretreatment, behavioral performance improved, hippocampal neuronal injury was alleviated, Nissl body loss was partially reversed, and HIF-1α expression was decreased, suggesting that BHD exerts protective effects against hypobaric hypoxia-induced neurological dysfunction and hippocampal structural damage (2, 37, 43).

HIF-1α is a key hypoxia-responsive molecule that facilitates oxygen supply and metabolic adaptation in the early stage of hypoxia, but persistent or excessive activation may contribute to brain edema, blood-brain barrier damage, neuronal injury progression, and inflammatory amplification (19–22). The PI3K-Akt pathway plays a central role in neuronal survival, inflammatory regulation, and neurovascular unit protection and may confer neuroprotection by inhibiting apoptosis, attenuating inflammatory responses, and maintaining blood-brain barrier homeostasis (23–27). In the present study, network pharmacology analysis showed that the common targets shared by BHD and high-altitude brain injury were significantly enriched in the HIF-1 signaling pathway, AGE-RAGE signaling pathway, and PI3K-Akt signaling pathway. Transcriptomic analysis further demonstrated that differential genes after hypobaric hypoxia exposure were significantly enriched in the PI3K-Akt signaling pathway, MAPK signaling pathway, ECM-receptor interaction, and antigen processing and presentation, whereas BHD-M intervention partially reversed these abnormalities. Notably, the PI3K-Akt signaling pathway was enriched in both network pharmacology and transcriptomic analyses, suggesting that this pathway may represent an important molecular basis for the protective effect of BHD against hypobaric hypoxia-induced brain injury.

RT-qPCR results further strengthened this mechanistic link. In the model group, the expression levels of HIF-1α, IL-6, NF-κB1, VEGFA, and STX1A in hippocampal tissue were increased, whereas BHD pretreatment reduced these expression levels to varying degrees. These findings suggest that hypobaric hypoxia-induced injury involves not only hypoxic responses and metabolic disturbance but also inflammatory amplification, vascular responses, and synaptic dysfunction. The ability of BHD to reverse these changes indicates that its neuroprotective effects are not restricted to a single pathway, but rather involve coordinated regulation of hypoxic responses, inflammatory reactions, vascular abnormalities, and synaptic dysfunction. In line with previous studies, BHD has been shown to alleviate ischemia-reperfusion-induced brain injury by suppressing oxidative stress and inflammation and to promote tissue repair by improving angiogenesis and microenvironmental homeostasis (39, 40). In addition, BHD has also been reported to regulate metabolic networks, promote neurogenesis, enhance synaptic plasticity, and improve neurological function in experimental models (13, 15–17, 41, 42). Therefore, the present findings support the notion that the neuroprotective effect of BHD is more likely achieved through coordinated modulation of HIF-1-related hypoxic responses and PI3K-Akt-related cell survival and repair signaling.

Metabolomics analysis provided additional support for this mechanism at the metabolic level. Significant changes were observed in serum levels of L-histidine, p-cresol sulfate, hyocholic acid, and glycocholic acid in the hypobaric hypoxia model group, suggesting that amino acid metabolism, microecological metabolites, and bile acid metabolism are involved in the pathological process of hypobaric hypoxia-induced brain injury (37, 38). Previous studies have shown that high-altitude or hypobaric hypoxia exposure can profoundly affect amino acid and energy metabolism networks and is often accompanied by oxidative stress, blood-brain barrier damage, and elevated inflammation (37, 38, 43, 44). In the present study, BHD significantly reversed the changes in these differential metabolites, indicating that its effect involves not only behavioral and histological improvement but also metabolic network remodeling. Overall, the abnormal energy metabolism revealed by metabolomics, the HIF-1/PI3K-Akt-related pathways identified by network pharmacology, the enrichment of the PI3K-Akt signaling pathway in transcriptomics, and the changes in hypoxia-, inflammation-, and synapse-related genes detected by RT-qPCR together form a relatively coherent evidence chain. These multi-layered findings suggest that BHD may alleviate hypobaric hypoxia-induced brain injury and promote functional recovery by coordinating HIF-1- and PI3K-Akt-related hypoxic responses, inflammatory regulation, cell survival, blood-brain barrier homeostasis, and metabolic regulation (39–44).

Several points require further interpretation. First, the immunofluorescence results showed increased hippocampal HIF-1α expression after hypobaric hypoxia and its reduction after BHD pretreatment, but the present staining was not combined with cell-type-specific markers. Therefore, the precise cellular sources of HIF-1α in this model, such as neurons, astrocytes, microglia/macrophages, endothelial cells, or other neurovascular-unit-associated cells, cannot be determined from the current data. Future double-immunofluorescence or single-cell approaches will be needed to clarify the cellular localization of HIF-1α and to determine how different hippocampal cell populations contribute to BHD-mediated neuroprotection.

Second, the responses of different hippocampal subregions were not completely identical. The CA2 region showed relatively limited recovery in the H&E and Nissl staining analyses compared with CA1, CA3, and DG. This may reflect region-specific vulnerability or repair capacity under hypobaric hypoxic stress, differences in local neuronal circuitry, or the limited sensitivity of the current histological quantification. Therefore, the weaker CA2 response should be interpreted cautiously and should not be regarded as evidence that BHD has no effect on this region.

Third, BHD and acetazolamide administration was initiated before hypobaric hypoxia exposure and continued during the 72 h exposure period; therefore, the present findings mainly support the preventive/prophylactic effect of BHD rather than a post-injury therapeutic effect. Whether BHD is effective when administered after the onset of hypobaric hypoxia-induced injury remains to be clarified. In addition, the present behavioral evaluation was mainly based on the open field test; motor coordination tests such as the rota-rod assay and cognitive paradigms such as the novel object recognition test were not performed. Therefore, the effects of BHD pretreatment on motor coordination and recognition memory under hypobaric hypoxia require further validation. Although each experimental group initially included eight mice, RT-qPCR validation was performed using four independent hippocampal samples from four individual mice per group because the remaining samples were allocated to histological, metabolomic, transcriptomic, and other validation assays. We acknowledge that n = 4 is relatively small for RT-qPCR analysis and may limit statistical power; therefore, future studies with larger sample sizes are needed to further validate these findings. The sample sizes used for immunofluorescence, histological quantification, and transcriptomic analysis were also relatively limited, although they were consistent with commonly used exploratory experimental designs. Additional studies with post-exposure therapeutic administration, longer observation periods, expanded behavioral testing, targeted metabolite validation, protein-level verification of key signaling molecules, and cell-specific mechanistic experiments are needed to further define the causal relationship among the identified metabolites, HIF-1/PI3K-Akt-related signaling, inflammatory responses, and neuroprotection.

## 5 Conclusions

**Figure 8:**
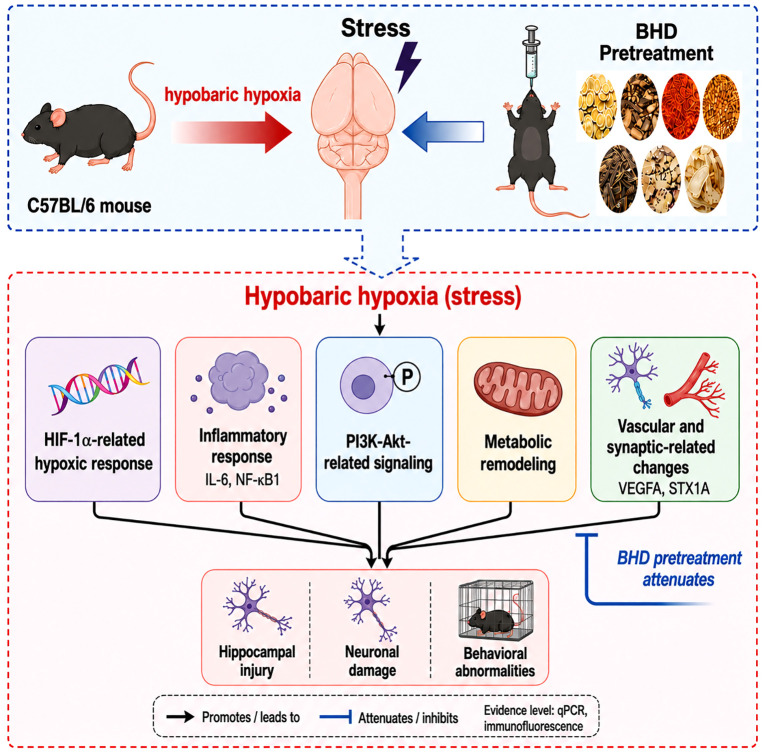
Proposed mechanism by which Buyang Huanwu Decoction protects against hypobaric hypoxia-induced brain injury. Hypobaric hypoxia may aggravate hippocampal injury, stress responses, inflammatory activation, and HIF-1-related hypoxic responses, whereas BHD pretreatment may alleviate these changes by coordinating metabolic remodeling, inflammatory regulation, and HIF-1/PI3K-Akt-related signaling.

This study demonstrated that BHD pretreatment prevented or attenuated hypobaric hypoxia-induced behavioral abnormalities and hippocampal injury in mice, as reflected by improved open-field performance, reduced neuronal and Nissl body loss, and decreased hippocampal HIF-1α expression. Integrated metabolomics, network pharmacology, transcriptomics, and RT-qPCR analyses suggest that the protective effect of BHD may be associated with coordinated regulation of metabolic remodeling, inflammatory responses, synaptic dysfunction, and HIF-1/PI3K-Akt-related signaling. These findings provide experimental evidence supporting the multi-component, multi-target, and multi-pathway protective potential of BHD against hypobaric hypoxia-induced brain injury, while further studies are needed to validate the causal roles of key metabolites and signaling targets (**Figure 8**).

## Data Availability

The metabolomics data presented in this study have been deposited in the MassIVE repository under accession number MSV000102105. The transcriptomic raw sequencing data have been deposited in the NCBI Sequence Read Archive (SRA) under BioProject accession number PRJNA1478120 and SRA study accession number SRP709346; the individual run accessions are SRR39138976-SRR39138984.

## Supplementary material

Supplementary Table 1 LC-MS/MS annotation information for representative differential metabolites identified in the non-targeted serum metabolomics analysis.

Supplementary Table 2 Primer sequences used for RT-qPCR analysis.

Supplementary Figure 1 Supporting metabolomics analyses, including correlation analysis, HMDB classification, LMSD classification, and KEGG classification of differential metabolites.

Supplementary Figure 2 Supporting transcriptomic enrichment analyses, including GO enrichment analyses for the CON vs. MOD and MOD vs. BHD-M comparisons and additional KEGG enrichment analysis for the MOD vs. BHD-M comparison.
