# Supplementary figures and images for: Protective effect and mechanisms of Buyang Huanwu decoction against hypobaric hypoxia-induced brain injury in mice: involvement of inflammatory responses and HIF-1/PI3K-Akt-related pathways
Source: Front Immunol. 2026 Jun 29;17:1856864. doi: 10.3389/fimmu.2026.1856864 (PMC13357224; doi:10.3389/fimmu.2026.1856864)

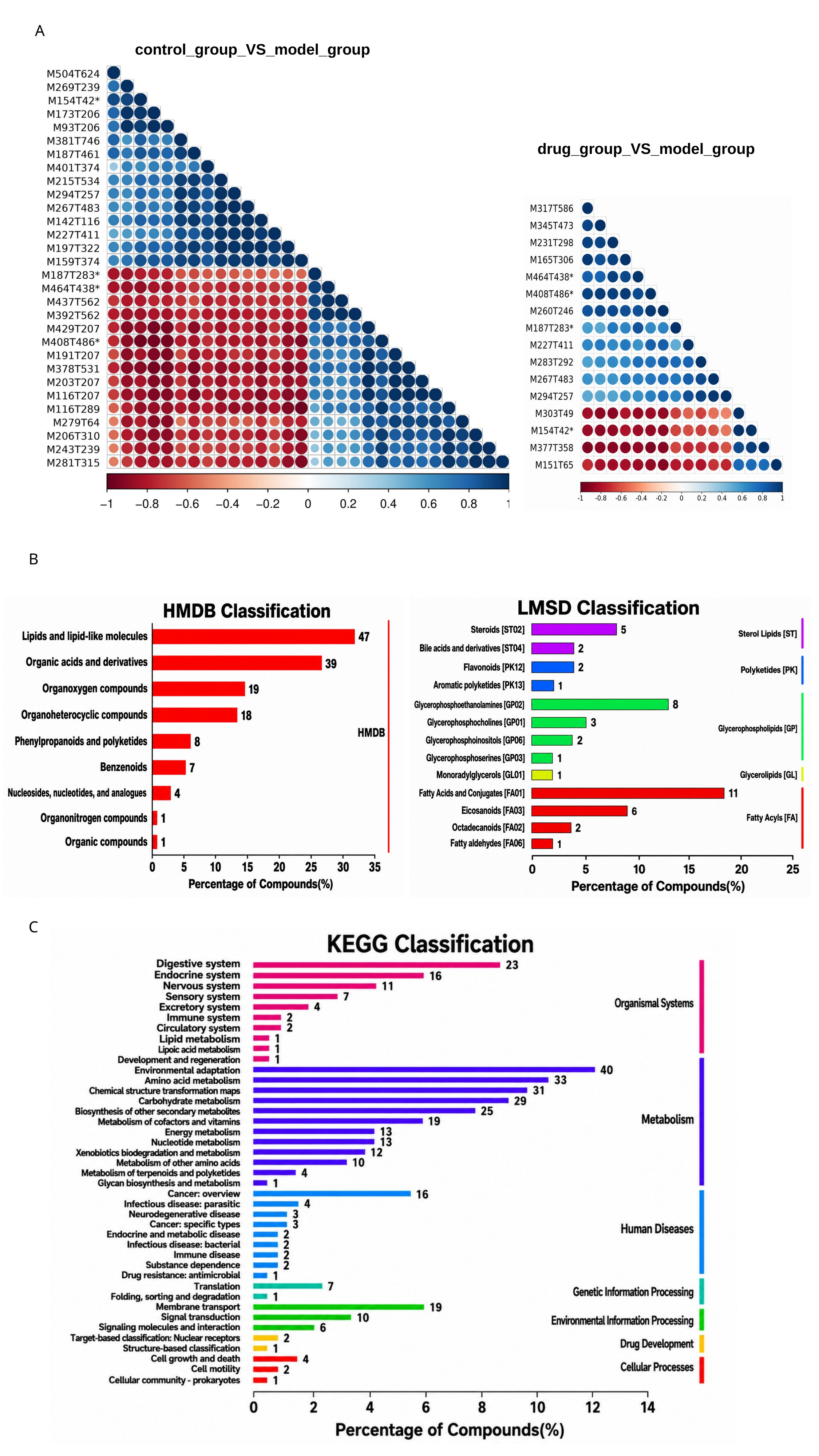

Supplement: Supplementary Figure 1 — Supporting metabolomics analyses, including correlation analysis, HMDB classification, LMSD classification, and KEGG classification of differential metabolites. [file Image1.tif]

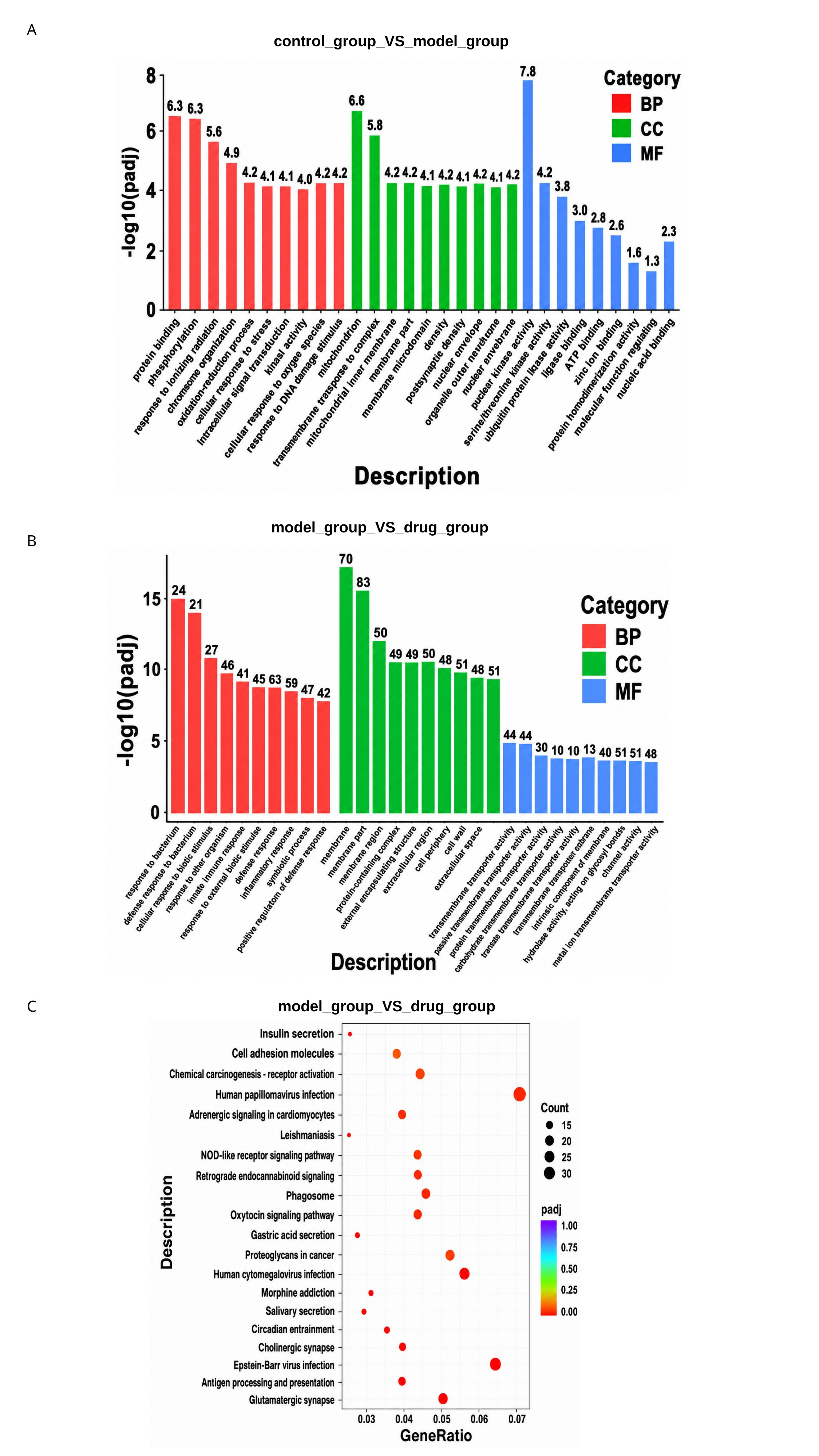

Supplement: Supplementary Figure 2 — Supporting transcriptomic enrichment analyses, including GO enrichment analyses for the CON vs. MOD and MOD vs. BHD-M comparisons and additional KEGG enrichment analysis for the MOD vs. BHD-M comparison. [file Image2.tif]
